# Maternal thyroid function test level during the first trimester of pregnancy at a center Yazd, Iran: A cross-sectional study

**DOI:** 10.18502/ijrm.v19i3.8573

**Published:** 2021-03-21

**Authors:** Somaye Gholami, Nasim Namiranian, Marzieh Shukohifar, Foroozandeh Kalantari, Reyhaneh Azizi

**Affiliations:** ^1^Diabetes Research Center, Shahid Sadoughi University of Medical Sciences, Yazd, Iran.; ^2^Deputy for Health Affairs, Shahid Sadoughi University of Medical Sciences, Yazd, Iran.

**Keywords:** First pregnancy trimester, Thyrotropin, Thyroxine, Triiodothyronine.

## Abstract

**Background:**

Thyroid dysfunction is associated with negative pregnancy outcomes. There is a lack of reliable information on thyroid hormones in Iranian pregnant women, especially in Yazd.

**Objective:**

To determine the safe thyroid hormone levels in women of Yazd and also the first trimester-specific reference ranges for serum thyroid-stimulating hormone (TSH), thyroxine (T4), and triiodothyronine (T3).

**Materials and Methods:**

In this cross-sectional study, the serum of 1,148 women in the first trimester of pregnancy was analyzed. Thyroid function tests (TSH, T4, T3) were measured through radioimmunoassay (RIA) [immunoradiometric (IRMA) for TSH]. The study was conducted in Yazd, from September 2018 to November 2019. Reference intervals were defined as the 5${}^{\mathrm{th}}$, 50${}^{\mathrm{th}}$, and 95${}^{\mathrm{th}}$ percentiles.

**Results:**

The mean age of the participants was 28.78 (± 5.86 yr) (range: 15-45 yr). The thyroid hormones reference intervals in the first trimester were TSH (0.2-3.8 mIU/l), T4 (7.45-12.75, μg/dl), and T3 (100-217 ng/dl).

**Conclusion:**

The results of the present study determined a local thyroid function measurement in the first trimester of pregnancy at a center of Yazd, Iran. This could facilitate the decision-making of maternal TSH level during the first trimester of pregnancy.

## 1. Introduction

There is an association between thyroid dysfunction in pregnancy, thyroid antibodies, and negative pregnancy outcomes. Obvious thyroid dysfunction is associated with numerous complications and consequences during pregnancy and postpartum (1). Maternal thyroid hormones are critical in fetal development, especially neural growth and development (1, 2). However, there is insufficient evidence regarding the association of subclinical thyroid disease during pregnancy with the risk of gynecological complications (3) and the preventive effects of levothyroxine therapy (4, 5). Diagnosis of subclinical hypothyroidism depends on the trimester of pregnancy and the reference intervals of thyroid tests and thyroid stimulating hormone (TSH) tests (6).

Moreover, the reference intervals for thyroid hormones depend on the ethnicity, iodine intake, body size, and the method of assessment (6). The National Academy of Clinical Biochemistry (NACB) recommends that reference intervals for TSH should be performed using samples of healthy individuals excluding those with known goiter and thyroid disease (7). Since there are no specific TSH references recommendation for the three trimesters of pregnancy for Yazd, Iran, the TSH reference range is based on American Thyroid Association (ATA) recommendations: 0.1-2.5 in the first, 0.2-3 in the second, and 0.3-3 in the third trimesters (8).

The World Health Organization recommends that epidemiological criteria can be used to evaluate the urinary iodine concentration (UIC) based on nutrition in pregnant women (9). During pregnancy, the median UICs between 150 µg/l and 249 µg/l define a population which has no iodine deficiency. Despite recent advances in biochemical methods and the ability to better understand the process of thyroid hormones in pregnancy, there are no reliable reference ranges in many countries, including some parts of Iran. Moreover, the findings of previous studies are inconsistent and cannot be generalized to our community. The cause of variation include: urinary iodine status, assay methods, and the inaccuracy in selecting the reference population and sample size.

The purpose of this study was the measurement of maternal TSH levels in the first trimester of pregnancy in Yazd, Iran.

## 2. Materials and Methods

### Study design and participants

This is cross-sectional study included all pregnant women who were referred to the primary care clinics of Shahid Sadoughi University of Medical Sciences in Yazd, Iran from September 2018 to November 2019.

There are 23 primary healthcare centers in Yazd city and 12 primary care clinics that were visited by pregnant women. For the purpose of this study, all pregnant women in first trimester, from September 2018 to November 2019, were screened. The information of 1,200 pregnant women in the first trimester of pregnancy was gathered. However, only 1,148 women who met the inclusion criteria were included.

The inclusion criteria were: Yazd-native pregnant women, in first trimester of pregnancy, age (15-45 yr). On the other hand, women with a history of thyroid disease, goiter, twin or multiple recurrent abortion, recurrent implantation failure, presence or history of thyroid nodules (on examination), and use of thyroid hormone replacement medication were excluded. The gestational age for thyroid hormone measurement (TSH, Thyroxine (T4), and Triiodothyronine (T3)) was considered as 9-13 wk. They were screened by thyroid hormone tests including TSH, T4, and T3.

All laboratory tests were done in the central laboratory approved by the Yazd Shahid Sadoughi University of Medical Sciences and Endocrinology and Metabolism as a reference laboratory. While the T4 and T3 were measured using the radioimmunoassay (RIA) method, TSH was measured by immunoradiometric (IRMA) for TSH using commercial kits (Izotop, Budapest, Hungray) and gamma counters (Wallac Wizard, Wallac Oy, Turku, Finland). The urinary iodine levels in the population of Yazd was measured in a pervious study on students with acceptable urinary iodine levels (18.82 ± 7.67 μg/dL). It can be concluded with this study that Yazd region has sufficient iodine intake (10).

### Ethical considerations

The study proposal was presented to the Ethics Committee of Shahid Sadoughi University of Medical Sciences and approved by the Internal Medicine Department. The Ethics Committee approved the study (code: IR.SSU.REC.1396.131). In addition, women were informed about the study and each participant provided written consent prior to the study.

### Statistical analysis

Data were analysed using the Statistical Package for the Social Sciences, version 18.0, SPSS Inc., Chicago, Illinois, USA (SPSS). The clinical and laboratory standards determined the normal and reference values. Additionally, Kolmogorov-Smirnov test was used to evaluate the normal distribution of thyroid hormones.

The frequency was used to describe qualitative variables and mean range, and standard deviation for quantitative variables. Analysis of variance was used to compare the mean thyroid hormones in different months of pregnancy. The study sample size was calculated according to the comparison of two means formula, considering type-one error: 0.05 and power: 0.8.

## 3. Results

**Figure 1:**
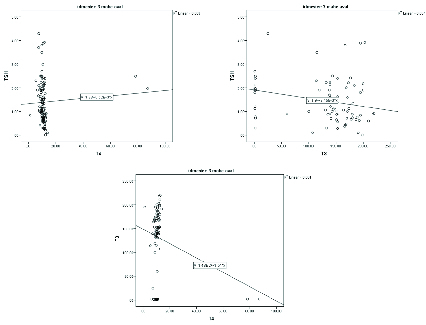
Scatter plot of correlation between (A) TSH and T4; (B) TSH and T3; and (C) T4 and T3.

The mean age of the study population (1,148 pregnant women) was 28.78 ± 5.86 yr. After applying the exclusion criteria, eligible pregnant women were included in the study. After applying the exclusion criteria, eligible pregnant women were included in the study. As presented in Table I, the mean level of TSH was 1.45 ± 0.97 mIU/l, T3 was 133.14 ± 17.08 ng/dl, and T4 was 11.95 ± 8.28 µg/dl. The first-trimester specific percentiles (5${}^{\mathrm{th}}$, 50${}^{\mathrm{th}}$, and 95${}^{\mathrm{th}}$) for TSH, T4, and T3, respectively, were based on the data of this study (Table I). Reference intervals in the first trimester were TSH (0.2-3.8 mIU/l), T4 (7.45-12.75, µg/dl), and T3 (100-217 ng/dl). No correlation was found between TSH and T4 or T3. Serum TSH had no significant correlation with T4 and T3. No significant correlation was found between the T4 and T3 in the first trimester (Figure 1A-1C).

**Table 1:** The Mean, range of thyroid hormones, and gestation-specific percentile values for TSH, T4, and T3


		**Confidence interval**		**Observed percentile**		
**Trimester**	Parameter*	Bias*	**Std. error**	**Lower**	**Upper**	**5${}^{\mathrm{th}}$**	**50${}^{\mathrm{th}}$**	**95${}^{\mathrm{th}}$**
				**TSH**	1.45 ± 0.97	0.0004 ± 0.0002	0.1092	1.23	1.67	0.28	1.13	3.8
**T4**	11.95 ± 8.28	0.0088 ± 0.0095	1.20	10.01	14.56	7.45	10.5	12.87
**T3**	133.14 ± 17.08	0.16 ± 0.084	8.32	113.34	148.75	100	153.6	217
*Data presented as Mean ± SD, TSH: Thyroid-stimulating hormone, T4: Thyroxine, T3: Triiodothyronine								

## 4. Discussion

Reference intervals in the first trimester were TSH (0.2-3.8 mIU/l), T4 (7.45-12.75, μg/dl), and T3 (100-217 ng/dl). Thyroid disorders in pregnancy are frequent and affect both the mother and fetus' health (11-13). There is a disagreement about the normal range of TSH in pregnancy and it is recommended that different populations determine their reference ranges. ATA reference range of TSH is 0.1-2.5 in the first trimester of pregnancy (8). In this study, we reported the first-trimester TSH, T3, and T4 levels in pregnant women in Yazd. In Gorgan study, with the similar methodology and different laboratory methods (14), the mean of TSH was 1.31 mIU/L (range, 0.1-6.2). It was reported to be 10% of the suppressive TSH population. In this study, enzyme-linked immunosorbent assay (ELISA), method was used to measure the TSH. In the present study, the first trimester of 5${}^{\mathrm{th}}$ and 95${}^{\mathrm{th}}$ percentiles TSH in this study were 0.28 and 3.8, respectively, which is similar to the TSH range of 0.2-3.9 reported in the study of Mehran and colleague (15). The present number is, however, higher than in other studies except for a few studies in India (16), United Arab Emirates (17), and China (18) where 2.5${}^{\mathrm{th}}$ and 97.5${}^{\mathrm{th}}$ percentiles were used and results for reference intervals with these laboratory kits are usually reported lower.

Many factors are affected in reporting reference ranges of thyroid hormones, such as maternal iodine levels, laboratory techniques, ethnicity, and methods of analysis. In our study, the TSH levels over 4 mIU/L have been reported for a limited number of individuals which may be due to screening of women before pregnancy and screening of hypothyroidism by endocrinologists in Yazd city. Subsequently, higher TSH is diagnosed earlier and many people are treated. In the present study, the T4 range was 7.5-12.8 µg/dl and the T3 range was 100-217 ng/dl in the 5${}^{\mathrm{th}}$ and 95${}^{\mathrm{th}}$ percentiles. In the study of Mehran colleague (15), the T4 range was reported as 7.5-12.8 µg/dl and the T3 range as 100-217 ng/dl in the 5${}^{\mathrm{th}}$ and 95${}^{\mathrm{th}}$ percentiles. Our results for T3 and T4 were inferior to that of Mehran L's study, which may be due to the differences in the laboratory method. The laboratory method of our study was RIA while in the study of Mehran and co-authors, it was isotope dilution tandem mass spectrometry (LC/MS/MS).

Furthermore, in the study of Kianpour and colleague (19), the TSH, T3, and T4 were measured by immunoassay and the mean levels of hormones reported were 1.84 ± 1.32 mIU/L, 1.01 ± 0.15 ng/dl, and 4.5 ± 0.64 pmol/L, respectively. Compared to our results, the mean TSH was approximately similar to that in the cited study. In the study of Soldin and colleague (20), using the same laboratory method like ours, the T3 and T4 ranges were reported as 92-218 ng/dl and 6.3-14.6 µg/dl, respectively. The strength of our study is the large number of cases that were performed for the first time in Yazd province. The limitations, on the other hand, were that the urinary iodine was not measured in pregnant women and the absence of data on anti-TPO thyroid autoantibodies.

## 5. Conclusion

The reference range in our study is close to the study of Mehran and colleague, which was conducted in Tehran (District 3). However, the ranges are higher than in European and American countries. Given that the iodine levels in the urine of pregnant women in Tehran are sufficient, this range may also be appropriate for pregnant women in Yazd.

##  Conflict of Interest

None declared.
